# Prostaglandin pathway gene expression in human placenta, amnion and choriodecidua is differentially affected by preterm and term labour and by uterine inflammation

**DOI:** 10.1186/1471-2393-14-241

**Published:** 2014-07-22

**Authors:** Robert J Phillips, Michel A Fortier, Andrés López Bernal

**Affiliations:** 1 Henry Wellcome Laboratories for Integrative Neuroscience and Endocrinology, School of Clinical Sciences, University of Bristol, Dorothy Hodgkin Building, Bristol BS1 3NY, UK; 2 Axe Reproduction, santé Périnatale et pédiatrie, Centre Hospitalier Universitaire de Québec (CHUL), Université Laval, 2705 boulevard Laurier, Ste-Foy, QC G1V 4G2, Canada; 3 St Michael’s Hospital, Southwell Street, Bristol BS2 8EG, UK

**Keywords:** Parturition, Inflammation, Pregnancy, Uterus

## Abstract

**Background:**

Elucidation of the biochemical pathways involved in activation of preterm and term human labour would facilitate the development of effective management and inform judgements regarding the necessity for preterm tocolysis and post-term induction. Prostaglandins act at all stages of human reproduction, and are potentially activators of labour.

**Methods:**

Expression of 15 genes involved in prostaglandin synthesis, transport and degradation was measured by qPCR using tissue samples from human placenta, amnion and choriodecidua at preterm and full-term vaginal and caesarean delivery. Cellular localisation of eight prostaglandin pathway proteins was determined by immunohistochemistry.

**Results:**

Expression of prostaglandin pathway genes was differentially affected by factors including gestational age at delivery, and the incidence and duration of labour. Chorioamnionitis/deciduitis was associated with upregulation of *PTGS2* (prostaglandin-endoperoxide synthase 2 (prostaglandin G/H synthase and cyclooxygenase)), along with the inflammatory genes *IL8* (interleukin 8), *S100A8* (S100 calcium binding protein A8) and *TLR2* (toll-like receptor 2), in amnion and choriodecidua, and with downregulation of *CBR1* (carbonyl reductase 1) and *HPGD* (hydroxyprostaglandin dehydrogenase 15-(NAD)) in choriodecidua. Protein localisation differed greatly between the various maternal and fetal cell types.

**Conclusions:**

Preterm and term labour are associated with distinct prostaglandin pathway expression profiles; inflammation provokes specific changes, unrelated to the presence of labour; spontaneous and induced term labour are indistinguishable.

## Background

Human labour requires a dramatic transition from a state of uterine quiescence and immune tolerance of the fetus—that prevails throughout pregnancy—to a brief period of intense uterine activation involving connective tissue remodelling and coordinated smooth muscle activity. The signals that initiate this process are not yet known, but among the candidates are the prostaglandins, which are known regulators of many aspects of reproductive physiology [1,2]. Evidence suggests that, during uterine activation there is positive feedback between prostaglandins and inflammatory cytokines that are released by infiltrating leukocytes [3]. Our early studies demonstrated that there is a relationship between inflammatory infiltration of the placenta, fetal membranes and decidua and increased prostaglandin and leukotriene release [4,5]. Inflammation has been associated with initiation of term and preterm labour both in the presence and absence of observable infection [6-12]. It is therefore possible that prostaglandins and inflammatory pathways are involved in uterine activation. It is important to establish the interactions between these pathways, both for women at risk of preterm birth who may be treated with anti-inflammatory drugs and prostaglandin synthesis inhibitors, and for women facing post-term induction of labour involving prostaglandin treatment.

**Figure 1:**
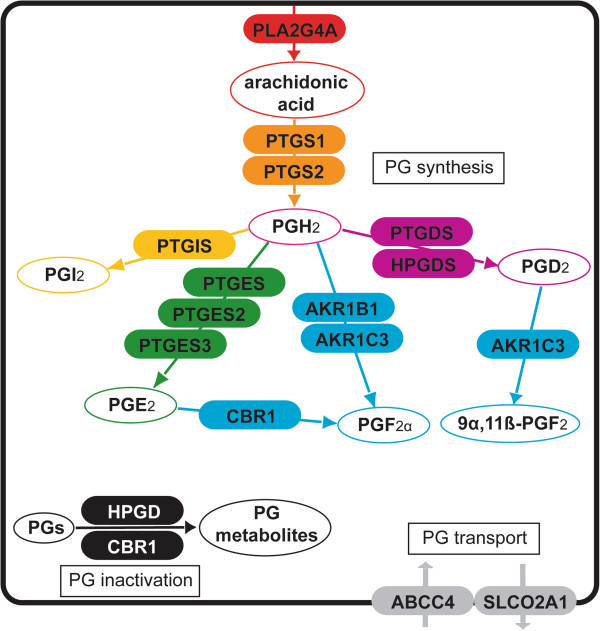
**Cellular pathways of prostaglandin (PG) metabolism.** A cell is depicted, showing enzymatic components (coloured boxes) involved in precursor prostaglandin synthesis, terminal prostaglandin synthesis, prostaglandin transport and prostaglandin inactivation, with reactions (thin arrows) and products (open circles).

We previously compared the relative levels of expression of 15 genes acting in all stages of prostaglandin metabolism (their relationships are illustrated in Figure 1) in human uterine tissues [13], demonstrating specific capacities for synthesis and catabolism of PGD_2_, PGE_2_, PGF_2_ and PGI_2_ in each tissue. We have now made a detailed examination of these genes in samples of placenta, choriodecidua and amnion, demonstrating that factors such as gestational age and the incidence and duration of labour are associated with significant changes in expression patterns. We have also characterised the distribution of prostaglandin pathway proteins throughout the constituent cells of the uterus using immunohistochemistry.

We have found distinct uterine prostaglandin gene expression and immunolocalisation in the presence of inflammation, suggesting uterine activation occurring through increased *PTGS2* expression in the fetal membranes and decreased degradative *HPGD* in the choriodecidua. Expression patterns in spontaneous preterm and term labour without inflammation differed from each other and from those with inflammatory changes. There were no differences between spontaneous and induced labour at term.

## Methods

### Collection of tissue

All women gave written informed consent according to the requirements of the North Somerset and South Bristol Research Ethics Committee. Placenta and gestational membranes were collected immediately post-partum from the following groups of women: preterm (25–36 weeks gestation) not-in-labour (PNIL), delivery by caesarean section for maternal or fetal complications; spontaneous preterm labour (SPL), with vaginal delivery; term (≥ 37 weeks gestation) not-in-labour (TNIL), delivery by elective caesarean section indicated by previous section and/or breech presentation; spontaneous term labour (STL), with vaginal delivery; term following induction of labour (IOL) with intravaginal PGE_2_ pessary and/or intravenous oxytocin infusion, with delivery vaginally or by emergency caesarean section (failure to progress). The women were of mixed parity and all delivered live singletons. None of the women in preterm labour received steroid treatment. Tissues were also collected from a group of women (INF) with evidence of inflammation, as suggested by clinical features of the women (pyrexia or uterine tenderness) and gross pathology of the delivered placentas, and confirmed histologically by the presence of leucocyte infiltration in the fetal membranes (chorioamnionitis), decidua (deciduitis) or placenta (intervillositis), with or without maternal pyrexia or uterine tenderness [4]. Clinical information for the women providing uterine tissues for this study is given in Table 1. Tissues from 36 women were used in this study; tissues from 31 of these women were previously among those used to study overall levels of prostaglandin pathway gene expression in placenta and gestational membranes [13]. Myometrial tissues used in the previous study were taken from a separate group of women. Gestational membranes were dissected from between 1 cm and 4 cm from the placental border. Placental tissue was dissected from > 5 mm beneath the maternal surface of the placenta. Tissue samples were dissected immediately after delivery (amnion and choriodecidua were separated by blunt dissection), washed in sterile phosphate-buffered saline (PBS), snap-frozen and stored in liquid nitrogen. Tissues were also fixed and paraffin-embedded following standard procedures for immunohistochemistry.

**Table 1:** Clinical information

**Mode of delivery**	**Number of women**	**Maternal age (years)**	**Gestational age at birth (weeks)**	**Duration of labour (hours)**	**Birthweight (kg)**	**Emergency: Elective Caesarean section**	**Membrane rupture (SRM:ARM)**	**Neonatal gender (male:female)**
PNIL	8	29 ± 9	33 ± 4	n/a	1.7 ± 0.7	2:6	n/a	2:6
SPL	5	27 ± 5	33 ± 1	4	2.0 ± 0.3	0:0	4:1	3:2
TNIL	7	31 ± 3	39 ± 2	n/a	4.0 ± 0.4	0:7	n/a	4:3
STL	6	31 ± 3	40 ± 1	4	3.6 ± 0.4	0:0	5:1	4:2
IOL	5	32 ± 9	40 ± 2	8	3.6 ± 0.5	1:0	3:2	5:0
INF	5	36 ± 7	32 ± 6	6	2.0 ± 1.3	2:0	3:2	4:1

Values are mean, mean ± standard deviation, or relative numbers in two groups. *Abbreviations*: *ARM* assisted rupture of the membranes, *INF* inflammation, *IOL* induction of labour, *PNIL* preterm not-in-labour, *SPL* spontaneous preterm labour, *SRM* spontaneous rupture of the membranes, *STL* spontaneous term labour, *TNIL* term not-in-labour.

### Quantitative real-time PCR (qPCR)

Total RNA was extracted from 100 mg tissue samples by the guanidine isothiocyanate/phenol method using 1 ml TRIzol (Invitrogen, Carlsbad, CA, US), giving yields of 10–150 μg. RNA was quantified using a GeneQuant II spectrophotometer (GE Healthcare, Little Chalfont, UK). 2 μg total RNA was used as a template for cDNA synthesis primed by random primers using the High Capacity cDNA Reverse Transcription Kit (Applied Biosystems, Foster City, CA, US). cDNA was diluted fourfold and 2 μl used as template for qPCR with the *Power* SYBR Green PCR Master Mix (Applied Biosystems), with reaction volume of 20 μl, forward and reverse primer concentrations of 75 nM, and 45 cycles of 95 C for 15 s and 60 C for 60 s, followed by a dissociation stage, using a 7500 Real-Time PCR System (Applied Biosystems). Two genes with least Ct variability, *POLR2A* (polymerase (RNA) II (DNA directed) polypeptide A, 220 kDa) and *ARHGDIA* (Rho GDP dissociation inhibitor (GDI) alpha), were chosen from five candidates for use as endogenous controls. PCR reaction efficiencies for all primer pairs were tested by serial template dilution, and were between 90% and 110%. The ‘sample maximization’ method was used, with reactions for each gene run on the minimum number of plates. A standard set of inter-run calibrators was included on each plate. Analysis was as previously described [13]. Sequences for all primers used in this study are given in Table 2.

**Table 2:** Primer sequences for quantitative real-time qPCR

**Gene**	**Accession**	**Forward primer**	**Reverse primer**
*PLA2G4A*	NM_024420	(205) AATGTCATTTATAGATCCTTACC	(486) GCATCCATTAACGTAATCTCC
*PTGS1*	NM_000962	(123) CAGCAGCCGCGCCATGAG	(355) ACAGGCCAGGGATGGTGC
*PTGS2*	NM_000963	(90) CTCAGACAGCAAAGCCTACC	(461) ATGTGATCTGGATGTCAACAC
*AKR1B1*	NM_001628	(71) AGCCATGGCAAGCCGTCTC	(317) GCACCACAGCTTGCTGACG
*AKR1C3*	NM_003739	(53) CAGACAAGTGACAGGGAATGG	(448) CCTCACCTGGCTTTAGAGAC
*CBR1*	NM_001757	(378) CCTGGACGTGCTGGTCAACA	(542) ACGTTCACCACTCTCCCTTG
*PTGES*	NM_004878	(50) AGAGATGCCTGCCCACAGC	(520) GCTGCTGGTCACAGGTGGC
*PTGES2*	NM_025072	(1354) ACTCAAGAGCAGGCACCGC	(1641) TGCCTTCCCTCTGCTCTGC
*PTGES3*	NM_006601	(29) GAGAAGTCGACTCCCTAGC	(305) TATGCTTGGAATCATTTGGATC
*PTGIS*	NM_000961	(46) AGCCCCGCGATGGCTTGG	(439) GAAGAGTCAGTTTCATCCTGG
*PTGDS*	NM_000954	(68) GCAGGAGAATGGCTACTCATC	(263) GACAACGCCGCCTTCTTCTC
*HPGDS*	NM_014485	(71) GACATAACACAGAATTGCACC	(280) CTGGTGAAGAGTAAGTCCATC
*HPGD*	NM_000860	(3) CTGCACCATGCACGTGAACG	(232) AAGTGTCTCTCAGTTGTTGCTG
*SLCO2A1*	NM_005630	(79) CAGCCATGGGGCTCCTGC	(328) GCATTGCTGATCTCATTCAAG
*ABCC4*	NM_005845	(3472) CAATCATACCTCAGGAACCTG	(3758) CTCATCAGTTCTTGGATCCAC
*ARHGDIA*	NM_004309	(358) ACCTGACGGGCGACCTGG	(628) GACTTGATGCTGTAGCTGCC
*POLR2A*	NM_000937	(4453) GCACCACGTCCAATGACATTG	(4719) GTGCGGCTGCTTCCATAAGC
*IL8*	NM_000584	(208) CTGTGTGAAGGTGCAGTTTTG	(344) GTGTTGGCGCAGTGTGGTC
*S100A9*	NM_002965	(233) GAGGACCTGGACACAAATGCA	(306) CAGGTTAGCCTCGCCATCAG
*TLR2*	NM_003264	(101) GAGACCTATAGTGACTCCCAG	(335) CTGCCCTTGCAGATACCATTG

Sequences written 5’-3’, with corresponding position of 5’-terminal nucleotide in mRNA indicated in parentheses. All sequences are from GenBank at NCBI.

### Immunohistochemistry

Slide-mounted, paraffin-embedded tissue sections were dewaxed in Histoclear (National Diagnostics, Atlanta, GA), hydrated in a graded ethanol series (absolute, 90%, 70% ethanol) and incubated in 1% (w/w) aqueous hydrogen peroxide solution for 15 min to block endogenous peroxidase activity. Antigen retrieval was achieved by incubation in citrate buffer (10 mM sodium citrate, pH6.0, 0.05% (v/v) Tween-20) at 95°C for 20 min. Slides were incubated for 20 min with 2% (v/v) blocking serum, washed with PBS and incubated overnight with primary antibody at the following dilutions: PTGS1 (prostaglandin-endoperoxide synthase 1 (prostaglandin G/H synthase and cyclooxygenase)) 1:60 (sc-1752, Santa Cruz Biotechnology, Santa Cruz, CA); PTGS2 1:60 (sc-1745); AKR1B1 (aldo-keto reductase family 1, member B1 (aldose reductase)) 1:200 (in house, Fortier MA); AKR1C3 (aldo-keto reductase family 1, member C3) 1:200 (ab27491, Abcam, Cambridge, UK); CBR1 1:300 (ab4148); PTGES (prostaglandin E synthase) 1:200 (160140, Cayman Europe, Tallinn, Estonia); HPGD 1:300 (in house, Fortier MA); SLCO2A1 (solute carrier organic anion transporter family, member 2A1) 1:3500 (in house, Fortier MA); VIM (vimentin) 1:200/1:1000 (V4630, Sigma, Gillingham, UK/M7020, DAKO, Ely, UK) in PBS + 3% (w/v) BSA. Slides were washed three times in PBS then incubated for 2 h with biotinylated secondary antibody (Vectastain ABC signal enhancement kit, Vector Labs, Burlingame, CA) diluted 1:200 in PBS + 3% (w/v) BSA. Slides were washed with PBS and incubated for 30 min with Vectastain ABC streptavidin-HRP (horseradish peroxidase) conjugate, washed again and incubated with 3,3’-Diaminobenzidine (DAB, Sigma) for antibody-specific colour development, which was stopped by washing in PBS, before counterstaining nuclei with Mayer’s Haemalum, dehydrating in a graded ethanol series followed by Histoclear and finally coverslip mounting using DPX mountant.

### Data analysis

Associations between levels of gene expression and continuous clinical variables (maternal and gestational age, duration of labour) were determined by measuring the probability of significance associated with the Pearson correlation coefficient (using the TDIST function in Excel). Comparisons of expression levels in distinct subgroups of subjects were made in Excel with Student’s t-tests (two-way, not assuming equal variances or equal sample size).

## Results

### Clinical correlations with PG gene expression

We investigated the possibility of relationships between clinical features of the subjects and prostaglandin gene expression levels in uterine tissues.

### Gestational age

**Figure 2:**
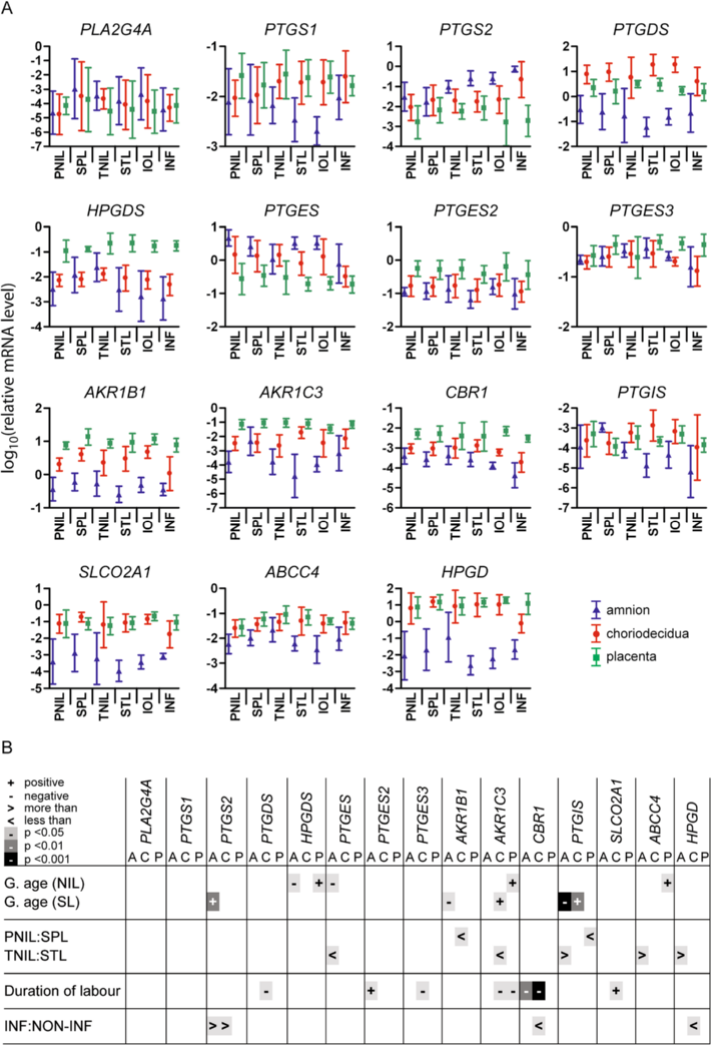
**Expression of prostaglandin pathway genes in pregnant human uterine tissues. (A)** Relative levels of mRNA by ΔΔCt method following qPCR, log_10_-transformed, shown as mean ± SD. A, amnion (blue); C, choriodecidua (red); P, placenta (green). PNIL, preterm not-in-labour; SPL, spontaneous preterm labour; TNIL, term not-in-labour; STL, spontaneous term labour; IOL, induction of labour; INF, inflammation. Numbers of samples: PNIL = 4; SPL = 4; TNIL = 6; STL = 5; IOL = 5; INF = 4. **(B)** Statistical comparisons of gene expression. Relationships with gestational age (g. age) in combined not-in-labour (NIL = PNIL + TNIL) and spontaneous labour (SL = SPL + STL) groups, and with duration of labour (SPL + STL + IOL) tested by correlation (Pearson’s); level of significance and direction of correlation are indicated. Comparisons between the presence and absence of labour (preterm and term) and inflammation were tested by Student’s t-tests.

Significant correlation between gestational age at delivery and prostaglandin gene expression occurred with gene and tissue specificity, as shown in Figure 2. In women who were not in labour at delivery, there was a negative correlation (decreasing gene expression with increasing gestational age) for *PTGES* in amnion (*p =* 0.045), and positive correlation for *HPGDS* (hematopoietic prostaglandin D synthase) in amnion (*p =* 0.039), *HPGDS*, *AKR1C3* and *ABCC4* in placenta (*p =* 0.020, 0.024, 0.046). In women delivering following spontaneous labour, there was negative correlation for *AKR1B1* and *PTGIS* (prostaglandin I2 (prostacyclin) synthase) in amnion (*p =* 0.049, < 0.001), and positive correlation for *PTGS2* in amnion (*p =* 0.007) and *AKR1C3* and *PTGIS* in choriodecidua (*p =* 0.026, 0.022). In these women, as expected, gestational age showed a strong positive correlation with birth weight (*p <* 0.001).

### Incidence of labour

Gene expression was compared between groups of women matched for gestational age who delivered with or without spontaneous labour. With preterm deliveries, expression was higher with labour for *AKR1B1* in choriodecidua and *PTGIS* in placenta (*p =* 0.032, 0.028). With term deliveries, expression was higher with labour for *PTGES* in amnion and *AKR1C3* in choriodecidua (*p =* 0.045, 0.033), while levels of *PTGIS*, *ABCC4* and *HPGD* in amnion were higher in deliveries without labour (*p =* 0.043, 0.049, 0.038).

### Duration of labour

Duration of labour in spontaneous and induced labour deliveries ranged from 33 minutes to 17 hours. Pearson correlation coefficients were calculated to determine the association between duration of labour and gene expression. Negative correlation, indicating decreasing expression with increasing duration, was seen with expression of *CBR1* in amnion (*p =* 0.006), *PTGDS* (prostaglandin D2 synthase 21 kDa (brain)), *PTGES3* (prostaglandin E synthase 3 (cytosolic)), *AKR1C3* and *CBR1* in choriodecidua (*p =* 0.049, 0.011, 0.013, <0.001) and *AKR1C3* in placenta (*p =* 0.031). Positive correlation was seen for *PTGES2* (prostaglandin E synthase 2) in amnion (*p =* 0.022) and *SLCO2A1* in choriodecidua (*p =* 0.010).

### Presence of inflammation

Placenta and gestational membranes were collected from women with uterine inflammation, and PG gene expression in this group was compared by t-test with expression in a subgroup of women with no inflammation that was matched for gestational age and mode of delivery (Figure 2). Effects of inflammation were limited to upregulation of *PTGS2* in amnion and choriodecidua (*p =* 0.022, 0.038), and downregulation of *CBR1* and *HPGD* in choriodecidua (*p =* 0.018, 0.011).

**Figure 3:**
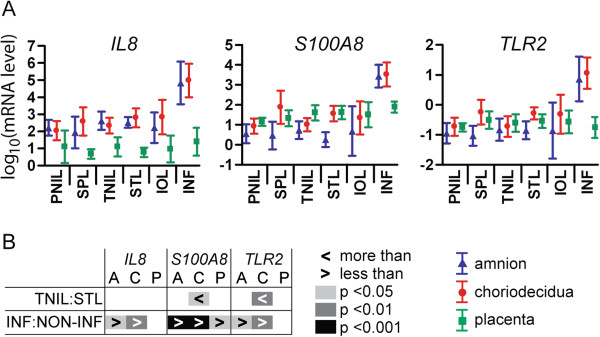
**Expression of inflammatory genes in pregnant human uterine tissues. (A)** Relative levels of mRNA by ΔΔCt method following qPCR, log_10_-transformed, shown as mean ± SD. PNIL, preterm not-in-labour; SPL, spontaneous preterm labour; TNIL, term not-in-labour; STL, spontaneous term labour; IOL, induction of labour; INF, inflammation. Numbers of samples: PNIL = 4; SPL = 4; TNIL = 6; STL = 5; IOL = 5; INF = 4. **(B)** Statistical comparisons of gene expression. No significant relationships were observed with gestational age in not-in-labour or spontaneous labour groups, between preterm and term not-in-labour or with duration of labour, so these comparisons are not shown. Comparisons of gene expression in the presence and absence of labour at term and of inflammation were tested by Student’s t-tests. Level of significance and direction of differential comparison are indicated. A, amnion; C, choriodecidua; P, placenta.

Women were assigned to the inflammation group on the basis of established histological criteria [4], and we further characterised the inflammatory status of all tissue samples by measurement of the expression of three genes known to be involved in inflammatory responses: *IL8*, *S100A8* and *TLR2* (Figure 3). All three genes were significantly upregulated in both amnion (*p* = 0.021, < 0.001, 0.012) and choriodecidua (*p* = 0.002, <0.001, 0.002) from women assigned to the inflammation (INF) group. In placenta, the only change was an increase in *S100A8* (*p* = 0.037) with inflammation. Both *S100A8* and *TLR2* were expressed at significantly higher levels in choriodecidua from women in the STL compared to the TNIL group (*p* = 0.014, 0.010) confirming a degree of inflammatory activity in term labour. Levels of both genes also appeared to be higher in SPL rather than PNIL choriodecidua, but these differences were of borderline significance (*p* = 0.061, 0.057).

### Immunolocalisation of PG pathway proteins in placenta

**Figure 4:**
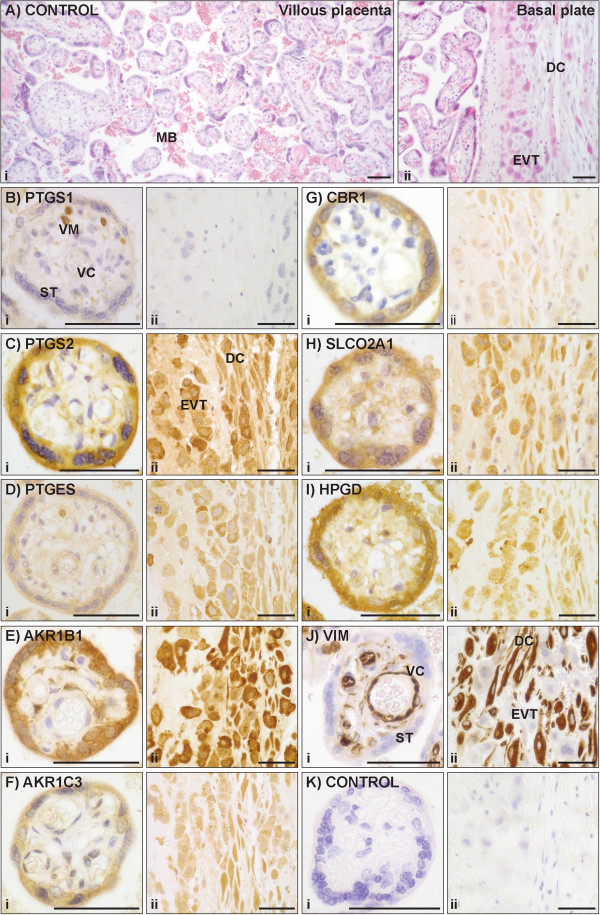
**Immunohistochemical localisation of PG pathway proteins in the placenta. (A)** H&E-stained control indicating structure of (i) placental villi, interspersed with maternal blood (MB), (ii) basal plate, containing extravillous trophoblasts (EVT) and decidual cells (DC). **(B-K)** Higher magnification images of (i) placental villi, indicating syncytiotrophoblasts (ST), vascular cells (VC) and villous macrophages (VM), (ii) basal plate. **(K)** Negative control without addition of primary antibody. Scale bar = 50 μm.

Low magnification images of H&E-stained placental sections in Figure 4A show (i) the fetal trophoblastic villi and intervillous space, which make up the great majority of the placenta, and (ii) the basal plate, which lies adjacent to the uterine wall. Figure 4B-I show placental immunolocalisation of eight of the PG pathway proteins, while Figure 4J shows the localisation of vimentin in villous fibroblasts, vascular cells, macrophages and decidual cells, but not trophoblasts.

In the chorionic plate (the surface of the placenta adjacent to the amniotic cavity), the amnion epithelium showed staining for PTGS2 and PTGES (not shown). Extravillous cytotrophoblasts, which form an incomplete layer at the inner border of the chorionic plate, showed staining for HPGD, PTGES, SLCO2A1, AKR1B1, AKR1C3 and CBR1.In the placental villi (Figure 4A-K(i)), syncytiotrophoblasts displayed staining for AKR1B1, HPGD PTGS2, SLCO2A1, CBR1, AKR1C3, and PTGES. Villous fibroblasts showed PTGS2 and SLCO2A1 staining and heterogeneous AKR1B1 staining. Villous macrophages were positive for PTGS1 and PTGES.

The basal plate of the placenta (Figure 4A-K(ii)) consists of maternal decidual cells and fetal extravillous cytotrophoblasts, in some areas arranged in distinct layers and in others partially or thoroughly interspersed. Both decidual cells and extravillous cytotrophoblasts showed staining for AKR1B1, PTGS2, HPGD, PTGES, SLCO2A1, AKR1C3, and CBR1. Staining in the two cell types varied from patient to patient and even in different regions of the same placental tissue section, notably with PTGES and HPGD in extravillous cytotrophoblasts. Extravillous cytotrophoblasts clustered in cell islands in the villous placenta had similar staining patterns (not shown). There was no noticeable staining for any of these proteins in fibrinoids of the basal plate (not shown). Protein distribution in the placental cell populations is summarised in Table 3, along with references to previous descriptions of these proteins.

**Table 3:** Immunolocalisation of PG pathway proteins in uterine cell populations

**Protein**	**PLACENTA**								**MEMBRANES**					
	**Basal plate**		**Chorionic Villi**				**Chorionic Plate**		**Choriodecidua**			**Amnion**		**INF**
	**EVT**	**DC**	**ST**	**VF**	**VM**	**VC**	**EVT**	**AE**	**DC**	**CT**	**CF**	**AF**	**AE**	**IL**
PTGS1			[14]	[15]	+[15]	[14]								
PTGS2	+[16]	+	+[14,16]	+[15]	[15,17]	[14]		+[18]	+[17,19]	+[19,20]	+[19]	+[19]	+[17,19,20]	+
PTGES	+[16]	+	+[21,22]		+	[21,22]	+	+		+[21-23]			+[21-23]	+
AKR1B1	+	+	+	+			+		+[21]	+[19]	+	+		
AKR1C3	+	+	+				+		+[21]	+[19]	+	+		+
CBR1	+	+	+				+		+	+	+	+		+
SLCO2A1	+	+	+	+			+		+[21]	+[19]				
HPGD	+[24]	+	+[18,24]				+		+[21]	+[18,19,24]	+	+		

Protein immunolocalisation identified in this study is represented by shaded cells; previous observations are referenced. *Abbreviations*: *AE* amniotic epithelium, *AF* amniotic fibroblasts, *CF* chorionic fibroblasts, *CT* chorionic trophoblasts, *DC* decidual cells, *EVT* extravillous trophoblasts, *IL* infiltrating leukocytes, *ST* syncytiotrophoblasts, *VC* vascular cells, *VF* villous fibroblasts, *VM* villous macrophages.

### Immunolocalisation of PG pathway proteins in gestational membranes

**Figure 5:**
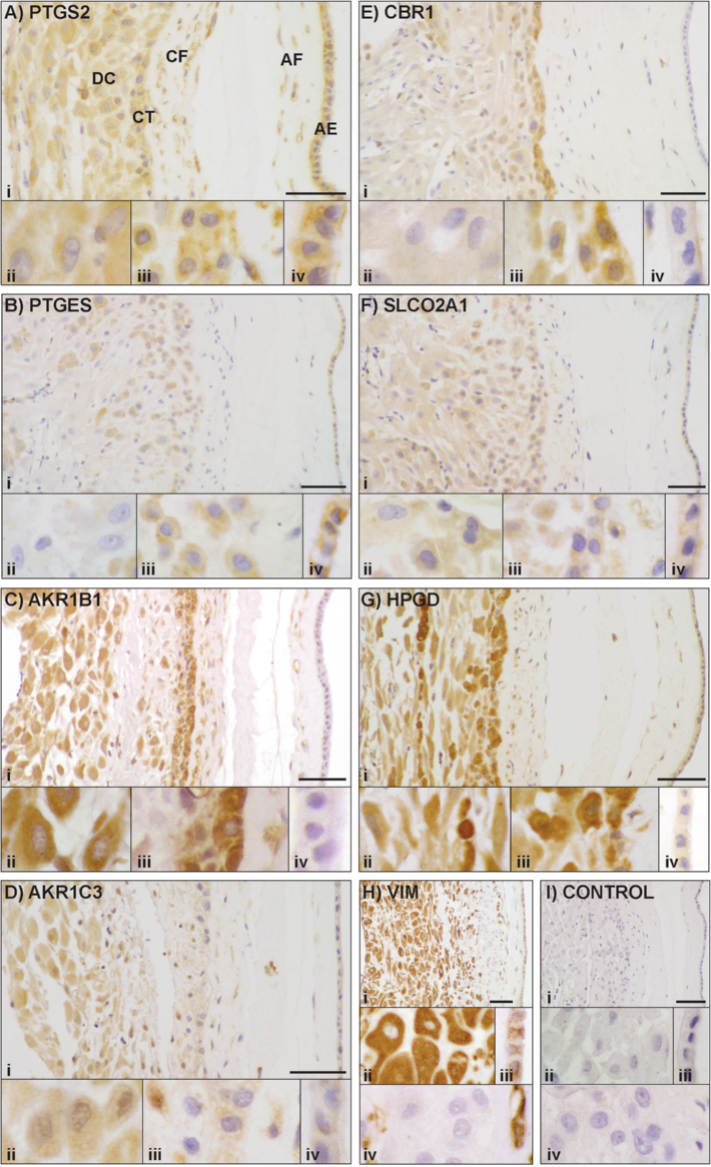
**Immunohistochemical localisation of PG pathway proteins in the gestational membranes. (A-I(i))** Lower magnification images show full thickness of membranes, containing amnion epithelium (AE), amnion fibroblasts (AF), chorionic fibroblasts (CF), chorionic trophoblast (CT) and decidual cells (DC). Higher magnification images show (ii) DC, (iii) CT, CF, (iv) AE. **(I)** Negative control without addition of primary antibody. Scale bar = 50 μm.

Figure 5A-G shows the immunolocalisation of seven of the PG pathway proteins in amnion and choriodecidua (PTGS1 is not included as we observed no staining in these tissues); Figure 5H shows vimentin localisation in decidual cells, amnion epithelium and fibroblasts of the amnion and chorion, but not in chorionic trophoblasts. In each panel a lower magnification image (i) gives a view through a full section of the membranes, while higher magnification images show (ii) decidual cells, (iii) chorionic trophoblasts and chorionic fibroblasts, (iv) amniotic epithelium.

The decidual cells showed staining for AKR1B1, HPGD, AKR1C3, PTGS2, SLCO2A1 and CBR1. Chorionic trophoblasts had staining for HPGD, AKR1B1, CBR1, PTGS2, PTGES, AKR1C3 and SLCO2A1. AKR1B1, PTGS2, AKR1C3, HPGD and CBR1 were seen in amniotic and chorionic fibroblasts. PTGS2 and PTGES had immunological reactions in amniotic epithelium. This protein distribution is summarised in Table 3.

### Influence of inflammation in fetal membranes on protein localisation

**Figure 6:**
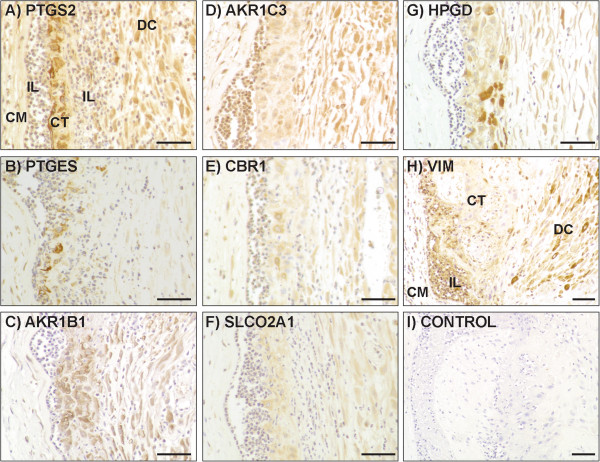
**Immunohistochemical localisation of PG pathway proteins in gestational membranes with inflammatory infiltration. (A-I)** Images show sections of membranes with chorionic fibroblasts (CF), infiltrating leukocytes (IL), chorionic trophoblast (CT) and decidual cells (DC). **(I)** Negative control without addition of primary antibody. Scale bar = 50 μm.

Inflammation results in disruption of the fetal membranes, with highly variable leukocytic infiltration and loss of integrity of the chorionic trophoblast layer. Within a tissue section it is common to see regions of massive infiltration with minimal remaining chorionic trophoblasts, alongside sections of membrane that appear relatively normal. Figure 6 shows immunolocalisation of prostaglandin proteins in membranes with a moderate inflammatory reaction, with considerable leukocytic infiltration but a relatively undiminished chorion. Prostaglandin pathway protein immunolocalisation in amniotic epithelium, amniotic and chorionic fibroblasts, and decidual cells was not noticeably altered by inflammation. In chorionic trophoblasts, heterogeneous expression of PTGS2, PTGES, CBR1 and HPGD was seen (Figure 6A, B, E & G). In inflammatory leukocytes there was expression of PTGS2, AKR1C3, CBR1 and PTGES (Table 3 and Figure 6A, B, D & E).

### Overlap with previous research

As we have examined multiple members of the prostaglandin pathway in three uterine tissues, there is inevitably a degree of overlap with previous studies of prostaglandin pathway components. For descriptions of the immunolocalisation of prostaglandin pathway proteins, this overlap has been summarised in Table 3, from which it can be seen that we are now presenting novel evidence of uterine immunolocalisation for seven of the eight prostaglandin pathway proteins studied.

Previous descriptions of prostaglandin pathway gene expression have focused largely on the cyclooxygenase/prostaglandin H2 synthase genes *PTGS1* and *PTGS2* (formerly Cox1 and Cox2). Not all previous observations can be reconciled with each other.

In the placenta, there is evidence suggesting no change in *PTGS1* expression with gestational age [15], and contrasting evidence of decreasing expression with increasing gestational age at labour [25]. In gestational membranes, increasing gestational age has been associated with increased [26,27], unchanged [27,28], and decreased [29]*PTGS1* expression. Likewise, the incidence of labour has been associated with increased [26,27] and unchanged [30-36]*PTGS1* expression.

In the placenta, the existing evidence suggests that there is no change in expression of *PTGS2* with gestational age or clinical chorioamnionitis [25]. In the gestational membranes, several studies have shown higher *PTGS2* expression with increasing gestational age [26-29]. There is evidence supporting both increased *PTGS2* expression following labour [26-28,31-35] and no change with labour [20,36,37].

Information relating to intrauterine expression of other prostaglandin pathway genes is limited. Our previous work demonstrated expression of the 15 prostaglandin pathway genes in placenta, amnion and choriodecidua [13]. In addition, *PLA2G4A* (phospholipase A2, group IVA (cytosolic, calcium-dependent)) expression has been identified in human placenta and gestational membranes [38], as has expression of *PTGDS* and *HPGDS*[39]. In placenta and membranes, *PTGES* expression has shown no change with labour [21]. Expression of *AKR1B1*, *AKR1C3*, *HPGD* and *SLCO2A1* has been demonstrated in amnion and choriodecidua [19]. Evidence has been presented in support of unchanged placental expression of *HPGD* in response to gestational age, labour and intrauterine infection [25,40], but also in support of increased expression with gestational age [41]. In choriodecidua, there is evidence for lower levels of *HPGD* mRNA in labour than not-in-labour [24,37,40,42], with further reductions occurring in the presence of intrauterine infection [40].

## Discussion

The human placenta, fetal membranes and decidua produce prostaglandins throughout pregnancy with a large increase at parturition, but the precise roles of these pleiotropic mediators are yet to be determined. The prostaglandin metabolic pathway consists of anabolic and catabolic components, as well as trans-membrane transporters (Figure 1). We have characterised prostaglandin pathway gene expression and protein localisation in placenta, amnion and choriodecidua from women delivered at different gestational ages with or without labour, induction and intrauterine inflammation. We have described novel protein localisation and gene expression patterns that increase our understanding of the roles of prostaglandins in human pregnancy and labour.

The placenta is the interface between the maternal and fetal blood supplies, allowing nutrient and waste exchange across the thin syncytiotrophoblast layers of numerous highly vascularised fetal villi projecting directly into the placental pool of maternal blood. As the fetal tissues are allogeneic to the maternal tissues, there must be mechanisms at this interface to prevent a maternal immune response to the fetus. We have identified similar patterns of protein localisation in decidual cells and extravillous trophoblasts of the placental bed and syncytiotrophoblasts of placental villi. These cells all express AKR1B1, PTGS2, HPGD, PTGES, SLCO2A1, AKR1C3 and CBR1, thus having the capacity for PGF_2_ and PGE_2_ synthesis and PG uptake and degradation. Gene expression patterns described here and in our previous work [13] support these observations and we now describe the presence of PGD_2_, PGE_2_ and PGI_2_ synthases in the placenta. Comparisons of placental gene expression in different groups of women identified increasing *HPGDS*, *AKR1C3* and *ABCC4* with gestational age in the absence of labour, and higher *PTGIS* in labour than not-in-labour preterm.

The fetal membranes consist of the fetal amnion and chorion and the attached maternal decidua, which together comprise a major structural element of the uterine tissues and have endocrine functions in pregnancy and parturition not yet fully elucidated [43]. As in the placenta, the trophoblast and decidua are the interface between maternal and fetal tissues. Immunolocalisation of prostaglandin pathway proteins in chorionic trophoblast cells and adjacent decidua are similar to each other, and to some extent resemble placental patterns, with HPGD, AKR1B1, AKR1C3, CBR1, PTGS2 and SLCO2A1 expressed in choriodecidua. Unlike in placental cells, variable protein expression is evident in choriodecidua, with the immunolocalisation of PTGES in chorionic trophoblast but not decidua, and higher chorionic levels of CBR1, and decidual levels of AKR1C3. Prostaglandin gene expression changes in choriodecidua include increased *AKR1C3* and *PTGIS* with gestational age and labour, with higher *AKR1B1* in labour preterm, and higher *AKR1C3* in labour at term compared with not-in-labour.

In the region between the chorionic trophoblast and amniotic epithelium, fibroblasts express PTGS2, PGF_2_ synthases and HPGD, while the amniotic epithelium itself, which is known to be a source of PGE_2_ synthesis [43,44], expresses PTGS2 and PTGES proteins, and also high levels of *PTGS2*, *PTGES* and *PTGES3* mRNA. Both *PTGS2* and *PTGES* are differentially expressed in amnion, with *PTGS2* increasing with gestational age in the presence of labour, and *PTGES* decreasing as gestational age rises in the absence of labour, and displaying higher expression in labour than not-in-labour at term. Despite previous observations of increased levels of prostaglandins and their metabolites in amniotic fluid with labour [39,45,46], we did not observe a significant alteration in *PTGS2* in amnion and choriodecidua with either preterm or term labour.

Taken together, these expression patterns suggest distinct roles for prostaglandin metabolism in tissues at the maternal:fetal interface and in tissues within the fetal compartment. At the interface there is the ability to synthesise PGD_2_, PGE_2_, and PGF_2_, but these prostaglandins might be limited to autocrine or paracrine function by the co-expressed degradative complex of SLCO2A1 and HPGD, which is considered to be a barrier between the maternal and fetal prostaglandin systems [24,47,48]. These prostaglandins could participate in the immunomodulation of maternal leukocytes present in decidua, placental bed and maternal blood, to prevent rejection of the fetal tissues.

PGE_2_ synthesised in the amnion and released into the amniotic fluid could influence fetal physiology, for example by inhibiting fetal breathing [49]. The reduction in amniotic *PTGES* expression and amniotic fluid PGE_2_[8] with increasing gestational age might then allow lung movements to develop in sync with fetal maturation. It should, of course, be noted that *PTGES* is the only one of the three PGE_2_ synthases that displays this dependence on gestational age for amniotic expression. *PTGES* is also the only PGE_2_ synthase that shows higher expression in the amnion than in the other tissues. Furthermore, as amniotic expression of both *SLCO2A1* and *HPGD* are some orders of magnitude lower than in placenta and choriodecidua, it suggests that there is sufficient degradation of the PGE_2_ that is released into the amniotic cavity in fetal tissues, such as the lung, to prevent accumulation in the amniotic fluid.

In addition to gestational age and the incidence of labour, we investigated the correlation of prostaglandin gene expression with other characteristics. Duration of labour was associated with different expression changes in each of the tissues, with both upregulation and downregulation of prostaglandin genes. The only gene to be affected by both duration of labour and the presence or absence of labour was *AKR1C3* in the choriodecidua. This suggests that regulation of some genes is associated with the process of labour, regardless of its duration, whereas others are affected by exposure to the prolonged stressful effects of labour. As we could not follow gene expression throughout labour, we cannot rule out that the differential regulation of these genes is a cause rather than an effect of the duration of labour. In a rarely quoted study involving >200 deliveries, Keski-Nisula *et al*. demonstrated that decidual inflammation is significantly more common in women in advanced labour compared to early labour, and concluded that the inflammatory changes are more likely to be a consequence of labour rather than its cause [50]. Given the traumatic effects of labour on both mother and child, elucidating the true nature of this relationship could provide valuable information.

We were very interested in evaluating the presence or absence of intrauterine inflammation. There has been a great deal of effort expended on establishing the causative relationship between intrauterine infection, inflammation and labour, particularly preterm labour. The premature activation of inflammatory pathways by intrauterine infection has been proposed as a major contributor to preterm labour [51,52]. Amniotic fluid metabolomic profiles differ in women delivering preterm in the presence and absence of intra-amniotic infection and inflammation [53].

We compared gene expression in a group of women with histological signs of inflammation with expression in a group of women matched for gestational age at delivery, and without substantial differences in other recorded variables, but with no signs of inflammation. To confirm the histological observations of inflammation, we measured the expression of three known inflammatory genes, finding significant upregulation of all three in amnion and choriodecidua samples from the INF group. Among the prostaglandin pathway genes, *PTGS2* was upregulated with inflammation in both amnion and choriodecidua, whereas *CBR1* and *HPGD* were downregulated in choriodecidua. In the placenta only one of the inflammatory control genes was upregulated, and none of the prostaglandin genes was affected by inflammation, but as the intrauterine inflammation was largely limited to chorioamnionitis/deciduitis, we cannot rule out that placentas affected by villitis, which show altered leukotriene synthesis [5], would also show prostaglandin pathway expression changes. The unique expression patterns of prostaglandin pathway and inflammatory control genes that we have observed suggest that in cases of uncomplicated spontaneous preterm labour, there is no underlying inflammatory expression profile. There must be an alternative mechanism for uterine activation in SPL in the absence of inflammation. In this regard it is worth mentioning that oxytocin, a strong uterotonic agent, stimulates *PTGS2* expression in human myometrial cells through previously undescribed pathways such as NFAT (nuclear factor of activated T cells) [54].

Although these results support the idea that labour usually occurs in the absence of inflammation, there is evidence that the presence of inflammation can be a trigger for labour, with [8,12] or without [10,12] signs of infection. This delivery mechanism can provide a response to intrauterine infections that can threaten the lives of mother and fetus. Tocolysis is not always an appropriate treatment, even for very early preterm labour, as the uterus can become a hostile environment. However, when infections can be overcome, and in instances of premature labour without infection and/or inflammation, there are great potential benefits to effective tocolysis. Our observation of different prostaglandin pathway expression profiles in preterm labour and inflammation could have implications for the choice of tocolytics used in different situations. Although elevation of *PTGS2* in placenta and membranes affected by inflammation could be countered by selective PTGS2 inhibitors, *PTGS2* is not upregulated with preterm labour in these tissues, although it is in myometrium [13]. Better understanding of the roles of PTGS2 in the different uterine tissues in preterm and term labour with and without inflammation could clarify when PTGS2 inhibitors are most likely to be effective.

We observed an increase in *PTGS2* expression in the amnion with term versus preterm labour that has also been seen previously [31,32,55]. An increase in amniotic fluid IL1 (interleukin 1) with labour at term has been described [56], and could be responsible for the *PTGS2* upregulation, although as with other observations in this field, there is contradictory evidence suggesting lower IL1 at term [8]. Increased *PTGS2* expression induced by cytokines, would explain the upregulation of *PTGS2* in the inflamed membranes of chorioamnionitis.

Limitations of this study include the numbers of samples in each of the groups; there is no enough data to correlate with previous preterm deliveries, hypertension, BMI, asthma, smoking and socioeconomic status of the women. Immunohistochemistry was used as a qualitative assay for only a subset of the prostaglandin pathway proteins, so that no quantitative data on protein levels were obtained. Another potential limitation is the lack of statistical correction for multiple comparisons, which could lead to type I errors of false positive identification of statistical significance. However, in order to avoid type II errors of rejection of true significance, we have presented the results of our statistical tests uncorrected, with the caveat that further studies are required before the changes that we have identified can be unequivocally confirmed.

## Conclusions

The principal aim of our research is to identify the causes of preterm labour, to enable reliable prediction of its occurrence and to facilitate its prevention by identifying biochemical pathways suitable for intervention. In light of considerable evidence linking prostaglandin function with uterine activation, we have undertaken a detailed analysis of prostaglandin pathway gene expression in human placenta, amnion and choriodecidua, identifying changes in association with gestational age, labour, inflammation and duration of labour, although there were no significant differences between spontaneous and induced labour at term. Inflammation provokes specific changes, unrelated to the presence of labour. The use of tocolytics should take into account these differences, in particular between uncomplicated spontaneous preterm labour and chorioamnionitis. Greater understanding of the different PG pathway changes in idiopathic and inflammation-associated preterm labour should facilitate the targeting of appropriate pharmacological intervention to these very different groups of women.

## Competing interests

The authors declare that they have no competing interest that could be perceived as prejudicing the impartiality of the research reported. MAF has a patent for methods for the regulation of the prostaglandin F synthase (PGFS) activity of AKR1B1 and uses thereof.

## Authors’ contributions

RJP: experimentation, analysis and manuscript preparation; MAF provided reagents helped with the preparation of manuscript; ALB: design of study and preparation of manuscript.

## Pre-publication history

The pre-publication history for this paper can be accessed here:

http://www.biomedcentral.com/1471-2393/14/241/prepub

## References

[B1] Challis JR Sloboda DM Alfaidy N Lye SJ Gibb W Patel FA Whittle WL Newnham JP Prostaglandins and mechanisms of preterm birth Reproduction 2002 124 1 17 10.1530/rep.0.1240001 12090913

[B2] Fortier MA Krishnaswamy K Danyod G Boucher-Kovalik S Chapdelaine JA A postgenomic integrated view of prostaglandins in reproduction: implications for other body systems J Phys Pharm 2008 59 Suppl 1 65 89 18802217

[B3] Christiaens I Zaragoza DB Guilbert L Robertson SA Mitchell BF Olson DM Inflammatory processes in preterm and term parturition J Reprod Immunol 2008 79 50 57 10.1016/j.jri.2008.04.002 18550178

[B4] López Bernal A Hansell DJ Khong TY Keeling JW Turnbull AC Prostaglandin E production by the fetal membranes in unexplained preterm labour and preterm labour associated with chorioamnionitis Br J Obstet Gynaecol 1989 96 1133 1139 10.1111/j.1471-0528.1989.tb03187.x 2590651

[B5] López Bernal A Hansell DJ Khong TY Keeling JW Turnbull AC Placental leukotriene B4 release in early pregnancy and in term and preterm labour Early Hum Dev 1990 23 93 99 10.1016/0378-3782(90)90132-3 2175261

[B6] López Bernal A Hansell DJ Cañete Soler R Keeling JW Turnbull AC Prostaglandins, chorioamnionitis and preterm labour Br J Obstet Gynaecol 1987 94 1156 1158 10.1111/j.1471-0528.1987.tb02315.x 2827724

[B7] Mueller-Heubach E Rubinstein DN Schwarz SS Histologic chorioamnionitis and preterm delivery in different patient populations Obstet Gynecol 1990 75 622 626 2314782

[B8] Hillier SL Witkin SS Krohn MA Watts DH Kiviat NB Eschenbach DA The relationship of amniotic fluid cytokines and preterm delivery, amniotic fluid infection, histologic chorioamnionitis, and chorioamnion infection Obstet Gynecol 1993 81 941 948 8497360

[B9] Opsjøn SL Wathen NC Tingulstad S Wiedswang G Sundan A Waage A Austgulen R Tumor necrosis factor, interleukin-1 and interleukin-6 in normal human pregnancy Am J Obstet Gynecol 1993 169 397 404 10.1016/0002-9378(93)90096-2 8362955

[B10] Steinborn A Günes H Röddiger S Halberstadt E Elevated placental cytokine release, a process associated with preterm labor in the absence of intrauterine infection Obstet Gynecol 1996 88 534 539 10.1016/0029-7844(96)00224-4 8841213

[B11] Leitich H Bodner-Adler B Brunbauer M Kaider A Egarter C Husslein P Bacterial vaginosis as a risk factor for preterm delivery: a meta-analysis Am J Obstet Gynecol 2003 189 139 147 10.1067/mob.2003.339 12861153

[B12] Shim SS Romero R Hong JS Park CW Jun JK Kim BI Yoon BH Clinical significance of intra-amniotic inflammation in patients with preterm premature rupture of membranes Am J Obstet Gynecol 2004 191 1339 1345 10.1016/j.ajog.2004.06.085 15507963

[B13] Phillips RJ Al-Zamil H Hunt LP Fortier MA López Bernal A Genes for prostaglandin synthesis, transport and inactivation are differentially expressed in human uterine tissues, and the prostaglandin F synthase AKR1B1 is induced in myometrial cells by inflammatory cytokines Mol Hum Reprod 2011 17 1 13 10.1093/molehr/gaq057 20595240

[B14] Xu Y Knipp GT Cook TJ Expression of cyclooxygenase isoforms in developing rat placenta, human term placenta, and BeWo human trophoblast model Mol Pharm 2005 2 481 490 10.1021/mp0500519 16323955

[B15] Wetzka B Nüsing R Charnock-Jones DS Schäfer W Zahradnik HP Smith SK Cyclooxygenase-1 and -2 in human placenta and placental bed after normal and pre-eclamptic pregnancies Hum Reprod 1997 12 2313 2320 10.1093/humrep/12.10.2313 9402302

[B16] Meadows JW Pitzer B Brockman DE Myatt L Differential localization of prostaglandin E synthase isoforms in human placental cell types Placenta 2004 25 259 265 10.1016/j.placenta.2003.09.004 15028417

[B17] Dunn-Albanese LR Ackerman WE 4th Xie Y Iams JD Kniss DA Reciprocal expression of peroxisome proliferator-activated receptor-gamma and cyclooxygenase-2 in human term parturition Am J Obstet Gynecol 2004 190 809 816 10.1016/j.ajog.2003.09.052 15042019

[B18] Cheung PY Walton JC Tai HH Riley SC Challis JR Immunocytochemical distribution and localization of 15-hydroxyprostaglandin dehydrogenase in human fetal membranes, decidua and placenta Am J Obstet Gynecol 1990 163 1445 1449 10.1016/0002-9378(90)90603-5 2240085

[B19] Breuiller-Fouché M Leroy MJ Dubois O Reinaud P Chissey A Qi H Germain G Fortier MA Charpigny G Differential expression of the enzymatic system controlling synthesis, metabolism, and transport of PGF2 alpha in human fetal membranes Biol Reprod 2010 83 155 162 10.1095/biolreprod.109.080390 20357271

[B20] Gibb W Sun M Localization of prostaglandin H synthase type 2 protein and mRNA in term human fetal membranes and decidua J Endocrinol 1996 150 497 503 10.1677/joe.0.1500497 8882169

[B21] Alfaidy N Sun M Challis JR Gibb W Expression of membrane prostaglandin E synthase in human placenta and fetal membranes and effect of labor Endocrine 2003 20 219 225 10.1385/ENDO:20:3:219 12721500

[B22] Premyslova M Li W Alfaidy N Bocking AD Campbell K Gibb W Challis JR Differential expression and regulation of microsomal prostaglandin E(2) synthase in human fetal membranes and placenta with infection and in cultured trophoblast cells J Clin Endocrinol Metab 2003 88 6040 6047 10.1210/jc.2003-030618 14671209

[B23] Meadows JW Eis AL Brockman DE Myatt L Expression and localization of prostaglandin E synthase isoforms in human fetal membranes in term and preterm labor J Clin Endocrinol Metab 2003 88 433 439 10.1210/jc.2002-021061 12519887

[B24] Sangha RK Walton JC Ensor CM Tai HH Challis JR Immunohistochemical localization, messenger ribonucleic acid abundance, and activity of 15-hydroxyprostaglandin dehydrogenase in placenta and fetal membranes during term and preterm labor J Clin Endocrinol Metab 1994 78 982 989 8157731 10.1210/jcem.78.4.8157731

[B25] Hirsch E Goldstein M Filipovich Y Wang H Placental expression of enzymes regulating prostaglandin synthesis and degradation Am J Obstet Gynecol 2005 192 1836 1842 10.1016/j.ajog.2004.12.070 15970823

[B26] Mijovic JE Zakar T Nairn TK Olson DM Prostaglandin endoperoxide H synthase (PGHS) activity and PGHS-1 and -2 messenger ribonucleic acid abundance in human chorion throughout gestation and with preterm labor J Clin Endocrinol Metab 1998 83 1358 1367 9543167 10.1210/jcem.83.4.4692

[B27] Mijovic JE Zakar T Angelova J Olson DM Prostaglandin endoperoxide H synthase mRNA expression in the human amnion and decidua during pregnancy and in the amnion at preterm labour Mol Hum Reprod 1999 5 182 187 10.1093/molehr/5.2.182 10065875

[B28] Slater DM Dennes WJ Campa JS Poston L Bennett PR Expression of cyclo-oxygenase types-1 and -2 in human myometrium throughout pregnancy Mol Hum Reprod 1999 5 880 884 10.1093/molehr/5.9.880 10460228

[B29] Johnson RF Mitchell CM Giles WB Bisits A Zakar T Mechanisms regulating prostaglandin H2 synthase-2 mRNA level in the amnion and chorion during pregnancy J Endocrinol 2006 188 603 610 10.1677/joe.1.06488 16522739

[B30] Freed KA Aitken MA Brennecke SP Rice GE Prostaglandin G/H synthase-1 messenger RNA relative abundance in human amnion, choriodecidua and placenta before, during and after spontaneous-onset labour at term Gynecol Obstet Invest 1995 39 73 78 10.1159/000292383 7737586

[B31] Hirst JJ Teixeira FJ Zakar T Olson DM Prostaglandin endoperoxide-H synthase-1 and -2 messenger ribonucleic acid levels in human amnion with spontaneous labor onset J Clin Endocrinol Metab 1995 80 517 523 7852513 10.1210/jcem.80.2.7852513

[B32] Slater DM Berger LC Newton R Moore GE Bennett PR Expression of cyclooxygenase types 1 and 2 in human fetal membranes at term Am J Obstet Gynecol 1995 172 77 82 10.1016/0002-9378(95)90087-X 7531399

[B33] Slater D Allport V Bennett P Changes in the expression of the type-2 but not the type-1 cyclo-oxygenase enzyme in chorion-decidua with the onset of labour Br J Obstet Gynaecol 1998 105 745 748 10.1111/j.1471-0528.1998.tb10205.x 9692415

[B34] Zakar T Olson DM Teixeira FJ Hirst JJ Regulation of prostaglandin endoperoxide H2 synthase in term human gestational tissues Acta Physiol Hung 1996 84 109 118 9046357

[B35] Mijovic JE Zakar T Nairn TK Olso DM Prostaglandin-endoperoxide H synthase-2 expression and activity increases with term labor in human chorion Am J Physiol 1997 272 E832 840 9176183 10.1152/ajpendo.1997.272.5.E832

[B36] Hirst JJ Mijovic JE Zakar T Olson DM Prostaglandin endoperoxide H synthase-1 and -2 mRNA levels and enzyme activity in human decidua at term labor J Soc Gynecol Investig 1998 5 13 20 10.1016/S1071-5576(97)00101-9 9501293

[B37] Makino S Zaragoza DB Mitchell BF Yonemoto H Olson DM Decidual activation: abundance and localization of prostaglandin F2alpha receptor (FP) mRNA and protein and uterine activation proteins in human decidua at preterm birth and term birth Placenta 2007 28 557 565 10.1016/j.placenta.2006.06.010 16911823

[B38] Freed KA Moses EK Brennecke SP Rice GE Differential expression of type II, IV and cytosolic PLA2 messenger RNA in human intrauterine tissues at term Mol Hum Reprod 1997 3 493 499 10.1093/molehr/3.6.493 9239738

[B39] Helliwell RJ Keelan JA Marvin KW Adams L Chang MC Anand A Sato TA O’Carroll S Chaiworapongsa T Romero RJ Mitchell MD Gestational age-dependent up-regulation of prostaglandin D synthase (PGDS) and production of PGDS-derived antiinflammatory prostaglandins in human placenta J Clin Endocrinol Metab 2006 91 597 606 10.1210/jc.2005-1982 16291703

[B40] van Meir CA Matthews SG Keirse MJ Ramirez MM Bocking A Challis JR 15-hydroxyprostaglandin dehydrogenase: implications in preterm labor with and without ascending infection J Clin Endocrinal Metab 1997 82 969 976 10.1210/jcem.82.3.3812 9062515

[B41] Schoof E Girstl M Frobenius W Kirschbaum M Repp R Knerr I Rascher W Dötsch J Course of placental 11beta-hydroxysteroid dehydrogenase type 2 and 15-hydroxyprostaglandin dehydrogenase mRNA expression during human gestation Eur J Endocrinol 2001 145 187 192 10.1530/eje.0.1450187 11454515

[B42] Pomini F Patel FA Mancuso S Challis JR Activity and expression of 15-hydroxyprostaglandin dehydrogenase in cultured chorionic trophoblast and villous trophoblast cells and in chorionic explants at term with and without spontaneous labor Am J Obstet Gynecol 2000 182 221 226 10.1016/S0002-9378(00)70516-3 10649182

[B43] Myatt L Sun K Role of fetal membranes in signaling of fetal maturation and parturition Int J Dev Biol 2010 54 545 553 10.1387/ijdb.082771lm 19924634

[B44] López Bernal A Newman GE Phizackerley PJR Turnbull AC Surfactant stimulates prostaglandin E production in human amnion Br J Obstet Gynaecol 1988 95 1013 1017 10.1111/j.1471-0528.1988.tb06506.x 3191038

[B45] Mitchell MD Keirse MJ Brunt JD Anderson AB Turnbull AC Concentrations of the prostacyclin metabolite, 6-keto-prostaglandin F1 alpha, in amniotic fluid during late pregnancy and labour Br J Obstet Gynaecol 1979 86 350 353 10.1111/j.1471-0528.1979.tb10609.x 380629

[B46] Lee SE Romero R Park IS Seong HS Park CW Yoon BH Amniotic fluid prostaglandin concentrations increase before the onset of spontaneous labor at term J Matern Fetal Neonatal Med 2008 21 89 94 10.1080/14767050701830514 18240075

[B47] Keirse MJ Turnbull AC Metabolism of prostaglandins within the pregnant uterus Br J Obstet Gynaecol 1975 82 887 893 10.1111/j.1471-0528.1975.tb00593.x 1191603

[B48] Nomura T Lu R Pucci ML Schuster VL The two-step model of prostaglandin signal termination: in vitro reconstitution with the prostaglandin transporter and prostaglandin 15 dehydrogenase Mol Pharmacol 2004 65 973 978 10.1124/mol.65.4.973 15044627

[B49] Sorokin Y Hallak M Klein O Kalderon I Abramovici H Effects of induction of labor with prostaglandin E2 on fetal breathing and body movements: controlled, randomized, double-blind study Obstet Gynecol 1992 80 788 791 1407917

[B50] Keski-Nisula L Aalto ML Katila ML Kirkinen P Intrauterine inflammation at term: a histopathologic study Hum Pathol 2000 31 841 846 10.1053/hupa.2000.8449 10923922

[B51] Goldenberg RL Hauth JC Andrews WW Intrauterine infection and preterm delivery N Engl J Med 2000 342 1500 1507 10.1056/NEJM200005183422007 10816189

[B52] Romero R Espinoza J Kusanovic JP Gotsch F Hassan S Erez O Chaiworapongsa T Mazor M The preterm parturition syndrome Br J Obstet Gynaecol 2006 113 Suppl 3 17 42 10.1111/j.1471-0528.2006.01120.x PMC7062298 17206962

[B53] Romero R Mazaki-Tovi S Vaisbuch E Kusanovic JP Chaiworapongsa T Gomez R Nien JK Yoon BH Mazor M Luo J Banks D Ryals J Beecher C Metabolomics in premature labor: a novel approach to identify patients at risk for preterm delivery J Matern Fetal Neonatal Med 2010 23 1344 1359 10.3109/14767058.2010.482618 20504069 PMC3440243

[B54] Pont JN McArdle CA López Bernal A Oxytocin-stimulated NFAT transcriptional activation in human myometrial cells Mol Endocrinol 2012 26 1743 1756 10.1210/me.2012-1057 22902539 PMC3507519

[B55] Fuentes A Spaziani EP O’Brien WF The expression of cyclooxygenase-2 (COX-2) in amnion and decidua following spontaneous labor Prostaglandins 1996 52 261 267 8936582 10.1016/s0090-6980(96)00088-3

[B56] Romero R Parvizi ST Oyarzun E Mazor M Wu YK Avila C Athanassiadis AP Mitchell MD Amniotic fluid interleukin-1 in spontaneous labor at term J Reprod Med 1990 35 235 238 2325034

